# Current status of insecticide resistance among malaria vectors in Kenya

**DOI:** 10.1186/s13071-017-2361-8

**Published:** 2017-09-19

**Authors:** Benyl M. Ondeto, Christopher Nyundo, Luna Kamau, Simon M. Muriu, Joseph M. Mwangangi, Kiambo Njagi, Evan M. Mathenge, Horace Ochanda, Charles M. Mbogo

**Affiliations:** 1 KEMRI, Centre for Geographic Medicine Research, Coast & KEMRI Wellcome Trust Research Programme, Kilifi, Kenya; 2 School of Biological Sciences, University of Nairobi, Nairobi, Kenya; 3 KEMRI, Centre for Biotechnology Research and Development, Nairobi, Kenya; 4 Department of Biological Sciences, Pwani University, Kilifi, Kenya; 5 Ministry of Health, Malaria Control Unit, Nairobi, Kenya; 6 KEMRI, Eastern and Southern Africa Centre of International Parasite Control, Nairobi, Kenya

**Keywords:** Insecticide resistance, Mechanism of resistance, *Anopheles*, Malaria, Kenya

## Abstract

**Background:**

Insecticide resistance has emerged as one of the major challenges facing National Malaria Control Programmes in Africa. A well-coordinated national database on insecticide resistance (*IRBase*) can facilitate the development of effective strategies for managing insecticide resistance and sustaining the effectiveness of chemical-based vector control measures. The aim of this study was to assemble a database on the current status of insecticide resistance among malaria vectors in Kenya.

**Methods:**

Data was obtained from published literature through PubMed, HINARI and Google Scholar searches and unpublished literature from government reports, research institutions reports and malaria control programme reports. Each data source was assigned a unique identification code and entered into Microsoft Excel 2010 datasheets. Base maps on the distribution of insecticide resistance and resistance mechanisms among malaria vectors in Kenya were generated using ArcGIS Desktop 10.1 (ESRI, Redlands, CA, USA).

**Results:**

Insecticide resistance status among the major malaria vectors in Kenya was reported in all the four classes of insecticides including pyrethroids, carbamates, organochlorines and organophosphates. Resistance to pyrethroids has been detected in *Anopheles gambiae* (*s*.*s.*), *An. arabiensis* and *An*. *funestus* (*s*.*s.*) while resistance to carbamates was limited to *An*. *gambiae* (*s*.*s.*) and *An*. *arabiensis.* Resistance to the organochlorine was reported in *An*. *gambiae* (*s*.*s.*) and *An*. *funestus* (*s*.*s.*) while resistance to organophosphates was reported in *An. gambiae* (*s*.*l.*) only. The mechanisms of insecticide resistance among malaria vectors reported include the *kdr* mutations (L 1014S and L 1014F) and elevated activity in carboxylesterase, glutathione S-transferases (GST) and monooxygenases. The *kdr* mutations L 1014S and L 1014F were detected in *An*. *gambiae* (*s*.*s.*) and *An. arabiensis* populations. Elevated activity of monooxygenases has been detected in both *An*. *arabiensis* and *An*. *gambiae* (*s*.*s.*) populations while the elevated activity of carboxylesterase and GST has been detected only in *An*. *arabiensis* populations.

**Conclusions:**

The geographical maps show the distribution of insecticide resistance and resistance mechanisms among malaria vectors in Kenya. The database generated will provide a guide to intervention policies and programmes in the fight against malaria.

**Electronic supplementary material:**

The online version of this article (10.1186/s13071-017-2361-8) contains supplementary material, which is available to authorized users.

## Background

Malaria remains a major public health concern worldwide and has a profound socio-economic impact on countries where it is endemic [1]. Globally, in 2015 an estimated 429,000 deaths from malaria occurred most of which were in children aged under 5 years in Africa [2]. Africa bears the greatest burden of malaria accounting for approximately 92% of malaria deaths [2].

Vector control remains one of the central components for malaria control through larval source reduction and adult vector control. The two main methods of adult malaria vector control are indoor residual spraying (IRS) and the use of insecticide-treated nets (ITNs) [3]. Twelve insecticide products are currently available for malaria vector control, confined to four chemical classes: pyrethroids, organochlorines, organophosphates, and carbamates. At present, only pyrethroids are approved for use in ITNs, the single most important malaria control intervention, responsible for averting approximately 68% of malaria deaths in Africa [4]. However, the widespread use of chemical insecticides in vector control programmes and agriculture has led to the development of insecticide resistance in many parts of Africa [5–9], threatening to reverse current gains in malaria control [10, 11].

Toxicity of pyrethroids and organochlorine [Dichlorodiphenyltrichloroethane (DDT)] to insects has been attributed to their activity on the nervous system. The voltage-sensitive sodium channel is the major target site for pyrethroids and DDT. Acetylcholine is the transmitter at central nervous system synapses in insects. For the nervous system to operate properly, it is necessary that, once the appropriate message has been passed, excess acetylcholine should be removed from the synapse, to prevent repetitive firing and to allow a succeeding message to be transmitted [12]. This removal is effected by the enzyme acetylcholinesterase (AChE1), which catalyses the hydrolysis of the ester bond. Organophosphate and carbamate insecticides inhibit the esterase, and the result is that acetylcholine accumulates in the synapses, so that nerve function is impaired which ultimately leads to the death of the insect [12].

Resistance mechanisms underpinning phenotypic resistance observed in malaria vectors have been described in the past and categorised into the target site, metabolic, behavioural resistance and reduced penetration of the insecticides [13–16]. One mechanism of altered target site resistance is mediated through knock-down resistance (*kdr*), involving point mutations in sodium channel genes in the mosquito’s nervous system resulting in cross-resistance to pyrethroids and DDT [17, 18]. The *kdr* mutation may lead to a substitution of leucine at locus 1014 of the sodium channel gene in the wild-type for phenylalanine resulting in L 1014F, or the leucine may be substituted with serine resulting in L 1014S [17, 18]. Another altered target site resistance involves mutations in the *ace-1* ^*R*^ gene resulting in insensitivity of acetylcholinesterase (AChE1) to carbamates and organophosphates. This mutation is caused by a single amino acid substitution, from a glycine to serine at the position 119, in the AChE1 catalytic site (G119S) [15, 16, 19]. In γ-aminobutyric acid (GABA)-mediated altered target site resistance, a single point mutation in the GABA receptor gene involving alanine-serine replacement in the heteromultimeric gated chloride ion channel results in resistance to dieldrin (*Rdl)* [20].

Metabolic resistance mechanisms are mediated by overexpression of metabolic enzymes by mosquito vectors in response to xenobiotic compounds. In response to selection pressure by organophosphates and carbamates, non-specific esterases (NSE) are overexpressed while DDT and pyrethroids leads to overexpression of cytochrome P450-dependent monooxygenases [21]. Glutathione-S-transferases (GST) elevation is significant in detoxification of organophosphates, DDT and pyrethroids [19]. The insect may produce increased quantities of these enzymes, which metabolise the insecticide, sequestrate the molecules so they cannot function and detoxification of the insecticide [16]. The over-expression of these enzymes may be as a result of gene amplification of the genes encoding these enzymes or changes in either trans-acting regulatory elements or the promoter region [19, 22].

Behavioural resistance was described by WHO in 1957 as ‘development of the ability to avoid a dose which would prove lethal’ [14] and is attributed to the irritant property of some insecticides which causes the malaria vectors to avoid sprayed surfaces. Studies have shown a shift in vectors biting preferences from indoor to outdoor and a change in behaviour from late night-biting to early night-biting to avoid insecticide-treated houses [23, 24].

Another important resistance mechanism is reduced penetration of insecticides which is mediated through cuticular thickening in insects leading to slower rates of insecticide absorption. This is an important mechanism when coupled with other mechanisms such as metabolic detoxification as it provides ample time for detoxifying enzymes to metabolise the chemical [13]. Studies have indicated that cuticular thickness is greater in pyrethroid resistant mosquitoes as compared to the pyrethroid susceptible mosquitoes [25].

In Kenya, the first reported case of resistance to pyrethroids in malaria vectors was in the context of insecticide-treated net use in western Kenya where reduced knockdown rates were observed [26]. Since then, widespread resistance to pyrethroid and DDT in malaria vectors have been reported in different parts of the country [9, 27–31]. Most studies in Kenya have focused on testing for *kdr* mutation in malaria vectors [31–35] with only a few studies focusing on metabolic resistance [36, 37] mainly due to difficulty in conducting metabolic tests [38]. Despite these difficulties, metabolic resistance requires further attention since it is likely to have more impact on the effectiveness of the insecticides than target-site resistance [38]. In addition, studies on target-site mechanism *ace-1* ^*R*^ gene are yet to be reported. Therefore, more studies need to be conducted urgently to provide a status update on presence or absence of this resistance mechanism in the *Anopheles* vectors and to monitor the spread of this resistance gene. This is crucial because carbamate and organophosphate insecticides have been suggested as a potential alternative to manage pyrethroid-resistant populations [8].

Several efforts have been made previously in Africa to generate databases on malaria vectors resistant to insecticides at national or continental level [18, 39–42]. These databases are crucial components in the monitoring, detection and management of insecticide resistance in vector species. In Kenya, studies on insecticide resistance to malaria vectors have increased over the years. Therefore, there is need to build an insecticide resistance database (*IRbase*) that will collate this data and enable policy makers to make rational decisions on proper and timely entomological and resistance monitoring that is evidence-based. The *IRBase* will be a geospatial database that will generate information essential to aid decision support systems to inform effective insecticide policy making by the Kenya National Malaria Control Programme.

The main aim of this study was to assemble a comprehensive Kenyan *IRBase* on the current status of insecticide resistance among malaria vectors in Kenya. The *IRBase* will be a valuable resource for use by the Kenya National Malaria Control Programme and other stakeholders involved in the monitoring and management of insecticide resistance.

## Methods

### Comprehensive literature search

A comprehensive search of online bibliographic databases of published literature was conducted to extract and create a database on the status of insecticide resistance among malaria vectors in Kenya (Table 1). The databases used included PubMed (http://www.ncbi.nlm.nih.gov/pubmed), HINARI (http://www.who.int/hinari/en/) and Google Scholar (http://scholar.google.com/). The key search terms formulated to guide scanning of published literature included *Anopheles,* insecticide bioassay, resistance, susceptible, susceptibility test, insecticide resistance mechanisms and Kenya. The unpublished literature was compiled from government reports, research institutions reports and malaria control programme reports (Table 1). The data extracted from unpublished sources had to adhere to standard WHO protocol thus a vigorous verification and authorization process was established [43]. The search period was confined to entomological surveys conducted between 1987 and 2015. This was deliberately done to ensure that the data collected included modern taxonomic species concepts such as cytological and molecular techniques which are capable of identifying sibling species within the *An. gambiae* and *An. funestus* complexes. The resulting literature was then reviewed retaining all references that met the following criteria for inclusion: (i) the reported study was undertaken after December 1986; (ii) the surveys reported primary data; (iii) the surveys provided study sites; (iv) the surveys reported the insecticide susceptibility tests or insecticides resistance mechanisms; and (v) the surveys adhered to the standard WHO protocol with the exception of permethrin in which different standard discriminating dosage (0.25%) applied from 1981 until 1998 [44, 45].

**Table 1:** Summary of a literature search for insecticide susceptibility and insecticide resistance mechanisms tests between 1994 and 2015, in Kenya

Reference or report source	Investigation type	Year of publication or report	Region	Study site	Data published in a journal
Kamau et al. [67]	Susceptibility; Mechanism	2007	Central; Coastal; Western	Susceptibility: Rota, Ahero Mechanism: Mwea, Kwale, Ahero, Kisii, Rota	Yes
Mathias et al. [31]	Susceptibility; Mechanism	2011	Western	Susceptibility: Asembo, Budalangi, Bungoma, Busia, Kakamega, Kisian, Malaba Mechanism: Asembo, Bungoma, Busia, Kakamega, Kisian, Malaba, Seme	Yes
Kawada et al. [27]	Susceptibility; Mechanism	2011	Western	Susceptibility: Akuot, Kibuogi, Mfangano, Ngou, Nyandago, Nyaroya, Takawiri, Uwi Mechanism: Mbita, Suba	Yes
Bonizzoni et al. [68]	Susceptibility; Mechanism	2012	Western	Susceptibility: Busia, Emutete, Ahero, Bungoma, Chulaimbo, Chemelil Mechanism: Busia, Emutete, Ahero, Bungoma, Chulaimbo, Chemelil	Yes
Kamau et al. [32]	Susceptibility	2006	Central	Mwea	Yes
Kawada et al. [28]	Susceptibility; Mechanism	2014	Western	Susceptibility: Nyamanga, Nyandago, Nyaroya, Hao, Roo, Rangwe, Mfangano Mechanism: Ragwe, Mfangano	Yes
Vulule et al. [69]	Susceptibility	1996	Western	Uriri, Uriri West, Ongoro-Ogero, Siala, Ojuach-Kanuto, Onyinyore-Othuthu	Yes
Vulule et al. [26]	Susceptibility	1994	Western	Uriri, Uriri West, Ongoro-Ogero, Siala, Ojuach-Kanuto, Onyinyore-Othuthu	Yes
Ochomo et al. [9]	Susceptibility	2014	Western	Bondo, Rachuonyo, Nyando, Teso	Yes
Mbogo et al. [70]	Susceptibility	1996	Coastal	Kilifi	Yes
Ochomo (unpub. obs., 2012, 2013, 2014)	Susceptibility	2012, 2013, 2014	Western	2012, 2014: Bondo, Rachuonyo, Nyando, Teso; 2013: Bondo, Bungoma, Gem, Homa Bay, Migori, Nyando, Rachuonyo, Teso	No
Chome (unpub. obs., 2010)	Susceptibility	2010	Coastal	Jego, Kinango, Magaoni	No
Orondo (unpub. obs., 2015)	Susceptibility	2015	Central; Coastal	Central: Murinduko, Karima, Kiamaciri; Coastal: Gwadu, Kidomaya, Marigiza	No
Msami (unpub. obs., 2012)	Susceptibility	2012	Coastal	Burangi, Jaribuni, Kimundia, Kiwalwa, Madunguni, Mavueni, Mbogolo, Shibe	No
Kiminza (unpub. obs., 2013)	Susceptibility; Mechanism	2013	Coastal	Susceptibility: Burangi, Kadzuhoni, Kaya-Dagamra, Mbogolo, Mkondoni Mechanism: Burangi, Kadzuhoni, Kaya-Dagamra, Mbogolo, Mkondoni	No
Munywoki (unpub. obs., 2013)	Susceptibility; Mechanism	2013	Coastal	Susceptibility: Burangi, Jaribuni, Kidutani, Mangororo, Mapawa, Mbogolo, Ngombeni, Shibe Mechanism: Burangi, Jaribuni, Kidutani, Mangororo, Mapawa, Mbogolo, Ngombeni, Shibe	No
KEMRI/ DOMC/ WHO (unpub. obs., 2012, 2013)	Susceptibility	2012, 2013	Western	2012: Bondo, Bungoma, Marindi, Muhoroni, Ndhiwa, Nyakach, Nyando, Nyatike, Rachuonyo, Rarieda, Rongo, Teso North, Teso South; 2013: Bondo, Bumula, Gem, Homabay, Karungu, Nyando, Rachuonyo, Teso, Teso North	No
Kawada et al. [34]	Mechanism	2011	Central; Coastal; Rift Valley; Western	Central: Kiambu, Kirinyaga, Kitui, Machakos, Makueni, Maragwa, Murangá, Nairobi, Nyeri, Thika; Coastal: Kilifi, Kwale, Lamu, Malindi, Mombasa, Taita, Tana River, Taveta; Rift Valley: Baringo, Kajiado, Koibatek, Samburu; Western: Bondo, Bungoma, Busia, Butere, Homa Bay, Kakamega, Kisumu, Lugari, Migori, Mumias, Nyando, Rachuonyo, Suba	Yes
Chen et al. [36]	Mechanism	2008	Western	Ahero, Asembo, Bondo, HomaBay, Iguhu, Kendo, Kibigori, Kisii, Kitale, Kombewa, Luanda, Lwanda, Miwani, Mumia, Rusinga, Stendmwako	Yes
Stump et al. [33]	Mechanism	2004	Western, Coastal	Western: Asembo, Kisian, Rota, Wathorego, Escarpment above Wathorego, Ahero, Nyakach, Muhoroni, Miwani; Coastal: Jego, Vaga	Yes
Vulule et al. [37]	Mechanism	1999	Western	Uriri, Uriri West, Ongoro-Ogero, Siala, Ojuach-Kanuto, Onyinyore-Othuthu	Yes
Ochomo (unpub. obs., 2011, 2012, 2013, 2014)	Mechanism	2011, 2012, 2013, 2014	Western	Bondo, Nyando, Rachuonyo, Teso	No
Mwangangi (unpub. obs., 2012)	Mechanism	2012	Coastal	Kiwalwa, Kimundia	No

### Data extraction and curation

The data were extracted into Microsoft Excel 2010 datasheets. From each published article and report, the information extracted from each susceptibility or mechanisms test conducted included: mosquito collection period (year, start month, end month), location name (province first administrative level, district second administrative level, village or site), GPS coordinates (latitude UTM_X, longitude UTM_Y), vector mosquitoes species (species or species complexes tested, stage tested and origin), data source (institute that collected data, data published in a journal or not, journal reference) and remarks indicating deviations from standard procedures. For bioassays following the WHO susceptibility testing protocol, the information recorded included: test type (WHO test kit-Adult), insecticide tested (pyrethroids, carbamates, organochlorine and organophosphates), bioassays conducted (number of replicates tested, total mosquito in all test replicates, number of replicates for control, total mosquitoes in all controls), and phenotypic test outcomes (time at which mortality recorded, recorded average mortality in treatment (%), recorded average mortality in controls (%), calculated average mortality adjusted for control (%), resistance status). The resistance status was based on recently revised WHO criteria: high, < 90%; moderate, 90–97%; and susceptible, 98–100% [43]. For biochemical or molecular mechanisms tests conducted, the following information was recorded: test type (*kdr* mutation- L 1014S and L 1014F, elevated activity of carboxylesterase, glutathione S-transferases and monooxygenases) and mechanism outcomes (mechanism status, allelic frequency in %).

### Geo-referencing of study sites

The study sites were geo-referenced using geographical coordinates provided in the research articles. Survey sites, whose geographical coordinates were not provided by the research articles, were geo-referenced using digital geographical databases, a collection of spatial data and related descriptive data organised for efficient storage and retrieval by many users, such as Microsoft Encarta, Google Earth (https://www.google.com/earth/) and Geonames (http://www.geonames.org/) [46].

### Generation of insecticide resistance maps and data summaries

Data from survey sites entered into Microsoft Excel 2010 datasheets were imported and converted to database (dbase) files used to generate spatio-temporal distribution maps in ArcGIS Desktop 10.1 (ESRI, Redlands, CA, USA). Spatial distribution of insecticide resistance status (susceptible, moderate or high) and mechanism status (detected or not detected) for each of the malaria vectors across Kenya by province were displayed.

The data extracted were summarised using Microsoft Excel 2010 datasheets. The data obtained from provinces in Kenya was summarized into the following regions: Western region (Western and Nyanza provinces); Central region (Central and Nairobi provinces); Eastern region (Eastern Province), Northeastern region (North Eastern Province); Coastal region (Coast Province) and the Great Rift Valley region (Rift Valley Province).

## Results

The search period between 1987 and 2015 yielded a total of 20 data articles (10 published and 10 unpublished) on insecticide susceptibility tests and 16 data articles (9 published and 7 unpublished) on insecticide resistance mechanisms that were identified and analysed (Table 2). The data articles that were obtained for this analysis were from 1994 to 2015 and none in the period between 1987 and 1993. The majority of data articles on insecticide susceptibility tests and insecticide resistance mechanisms occurred between 2009 and 2013.

**Table 2:** Some publications and reports from which data were extracted on insecticide susceptibility and resistance mechanisms between 1994 and 2015, in Kenya

	1994–1998	1999–2003	2004–2008	2009–2013	2014–2015	Total
Insecticide susceptibility data						
Published data	3	0	2	3	2	10
Unpublished data	0	0	0	8	2	10
Total	3	0	2	11	4	20
Insecticide resistance mechanism data						
Published data	0	1	3	4	1	9
Unpublished data	0	0	0	6	1	7
Total	0	1	3	10	2	16

Table 3 shows spatially unique survey sites reporting data for *Anopheles* species tested against the four classes of insecticides and the mechanisms of resistance in the regions of Kenya. Literature searches generated a total of 816 insecticide susceptibility data points and 473 insecticide resistance mechanisms data points of *Anopheles* species tested via recommended WHO methods [43]. Majority of the data points on insecticide susceptibility tests were from Western (82.7%, *n* = 675) and Coastal regions (15.4%, *n* = 126). Most studies (76.6%, *n* = 625) had tested resistance against pyrethroids mainly permethrin and deltamethrin. Few studies (23.4%, *n* = 191) focused on organophosphates (fenitrothion and malathion), carbamates (bendiocarb and propoxur) and organochlorines mainly DDT. However, a few studies conducted susceptibility tests in a study site more than once over the years for a given insecticide. Majority of the data points on resistance mechanisms were from Western (70.2%, *n* = 332) and Coastal regions (15.2%, *n* = 72). These studies had investigated mostly target-site resistance (88.6%, *n* = 419) (*kdr* mutations- L 1014S and L 1014F) and very few studies on metabolic resistance (11.4%, *n* = 54) including elevated activity of monooxygenases, carboxylesterase and glutathione S-transferases.

**Table 3:** Spatially unique survey sites by region that report data for *Anopheles* species that were tested against the four classes of insecticides using WHO insecticide susceptibility tests and insecticide resistance mechanisms tested using standard biochemical or molecular methods between 1994 and 2015, in Kenya

	Central	Coastal	Western	Eastern	The Great Rift Valley
Insecticide class					
Pyrethroids	9	64	552	–	–
Organophosphates	1	21	47	–	–
Carbamates	1	20	47	–	–
Organochlorines	4	21	29	–	–
Mechanism type					
*kdr* L1014S	14	46	256	8	13
*kdr* L1014F	9	20	40	6	7
Monooxygenases	4	6	28	2	6
Carboxylesterase	–^a^	–	4	–	–
Glutathione S-transferases	–	–	4	–	–

^a^“–”, test not done

**Fig. 1:**
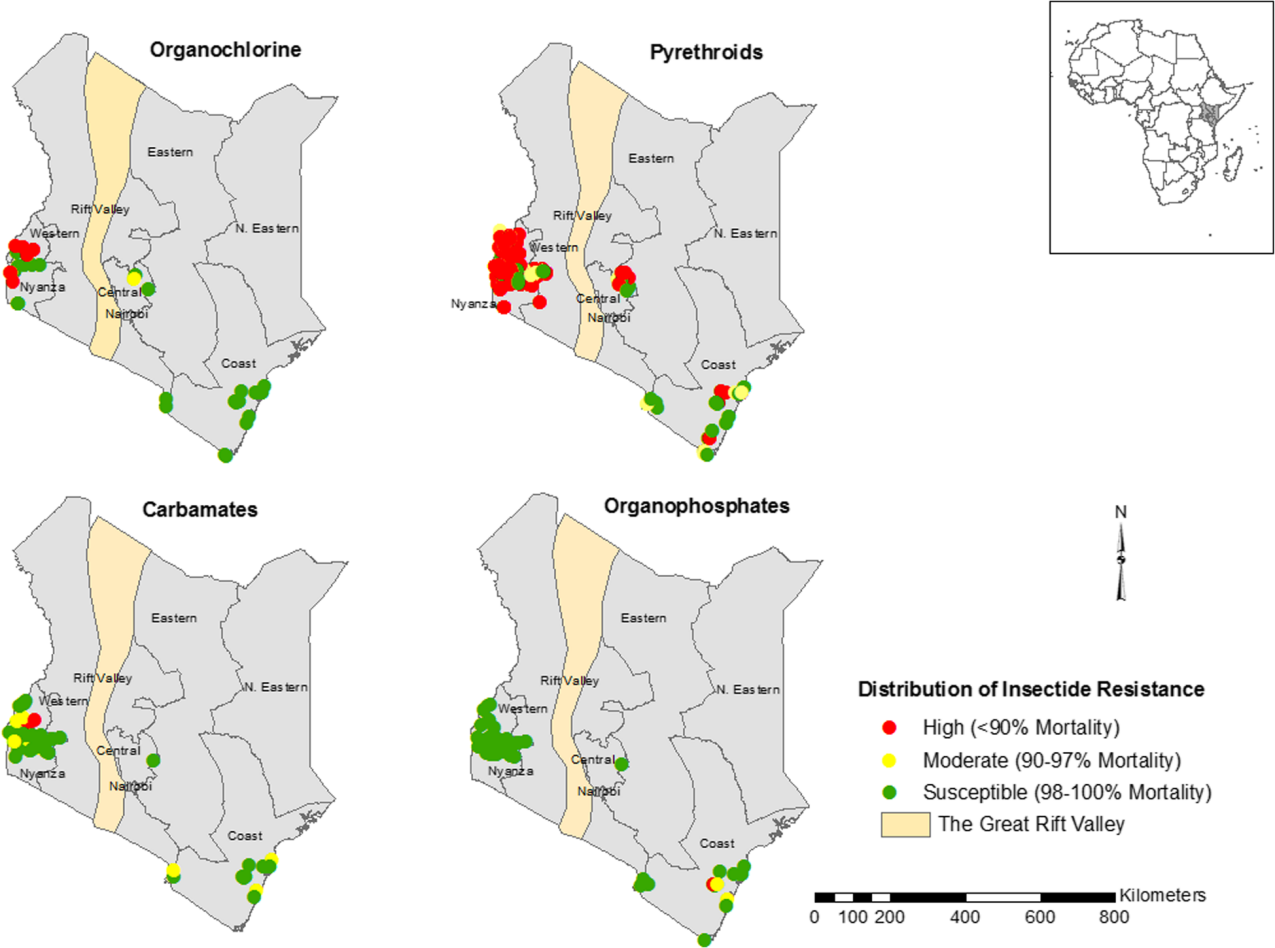
Distribution of insecticide resistance in *Anopheles* species tested between 1994 and 2015. *Anopheles* species that were tested were mainly resistant to pyrethroids across most regions of the country. Susceptibility to organophosphates, organochlorine and carbamates in *Anopheles* species tested was mainly observed with a few cases of resistance reported in Kenya

A summary of different classes of insecticides tested against malaria vectors in Kenya showed the differential level of response (Table 4). Pyrethroid and DDT resistance in the *Anopheles* species tested ranged from complete susceptibility to very high levels of resistance (Table 4). *Anopheles arabiensis* showed low frequency of pyrethroid resistance in Coastal and Central regions (Additional file 1: Figures S1- S3; Table 4) and also remained susceptible to DDT in the regions tested (Additional file 1: Figure S6; Table 4). The *An*. *funestus* species showed high resistance to pyrethroids in Western and Coastal regions whereas, in the Central region, it showed susceptibility (Additional file 1: Figures S1, S2; Table 4). Across all regions, the *Anopheles* species tested demonstrated high pyrethroid resistance, although some sites in Coastal and Central regions had low to moderate resistance (Fig. 1; Additional file 1: Figures S1-S5). These *Anopheles* species were largely susceptible to organochlorine (DDT) across most regions of Kenya with a few cases of high resistance reported in the western region (Fig. 1; Additional file 1: Figure S6).

**Table 4:** Mean % mortality of *Anopheles* species that were tested against the four classes of insecticides using WHO insecticide susceptibility tests between 1994 and 2015, in Kenya

	Pyrethroids					Organophosphates		Carbamates		Organochlorines
	Alphacypermethrin	Permethrin	Deltamethrin	Lambda-cyhalothrin	Etofenprox	Fenitrothion	Malathion	Bendiocarb	Propoxur	DDT
Central										
*An. arabiensis*	–	100	–	99.6	–	100	–	100	–	100
*An. gambiae* (*s.l*.)	–	55.4	74.2	–	–	–	–	–	–	98.4
*An. funestus* (*s.l*.)	–	93.6	100	–	–	–	–	–	–	100
Coastal										
*An. arabiensis*	–	100	100	–	–	97	–	97.7	–	100
*An. gambiae* (*s.s*.)	–	97.3	98.7	–	–	100	–	–	–	100
*An. gambiae* (*s.l*.)	–	92.5	91.3	96.5	–	98.9	–	98.8	–	99.7
*An. funestus* (*s.l*.)	–	91.1	86.2	–	–	–	–	–	–	–
Western										
*An. arabiensis*	83	66	79.9	53.5	82.3	100	99.9	98.3	100	99.8
*An. gambiae* (*s.s*.)	–	59.9	68.2	–	–	100	100	95.2	95	54.1
*An. gambiae* (*s.l*.)	70.2	73.8	70.4	55.5	–	100	99.7	100	–	67.9
*An. funestus* (*s.s*.)	– ^a^	28.9	72.9	–	–	100	–	–	100	94.4
*An. funestus* (*s.l.)*	–	99.7	43.5	100	–	100	–	100	–	100

^a^“–”, test not done

WHO criteria for resistance status applied: < 90%, high; 90–97%, moderate; 98–100%, susceptible

Against organophosphates, the majority of the *Anopheles* species tested showed susceptibility to these insecticides (Table 4; Fig. 1). However, a few cases of moderate resistance to fenitrothion were reported in the Coastal region and high resistance in *An*. *gambiae* (*s.l*.) reported in one site in the Coastal region (Additional file 1: Figure S7). Susceptibility to malathion was only tested for mosquitoes in the Western region of Kenya, and all *Anopheles* species tested remained susceptible to malathion in the Western region where it was only tested (Additional file 1: Figure S8).

Carbamates susceptibility in the *Anopheles* species tested varied from susceptible to moderate resistance (Table 4). The majority of the *Anopheles* species showed susceptibility to carbamates in all the regions of Kenya tested (Fig. 1) although *An*. *gambiae* (*s.s*.) and *An*. *arabiensis* were resistant to bendiocarb at two sites in the Western region (Additional file 1: Figure S9). Moderate level of resistance against carbamates was also reported in the *Anopheles* species tested in Western and Coastal regions (Additional file 1: Figures S9, S10).

**Fig. 2:**
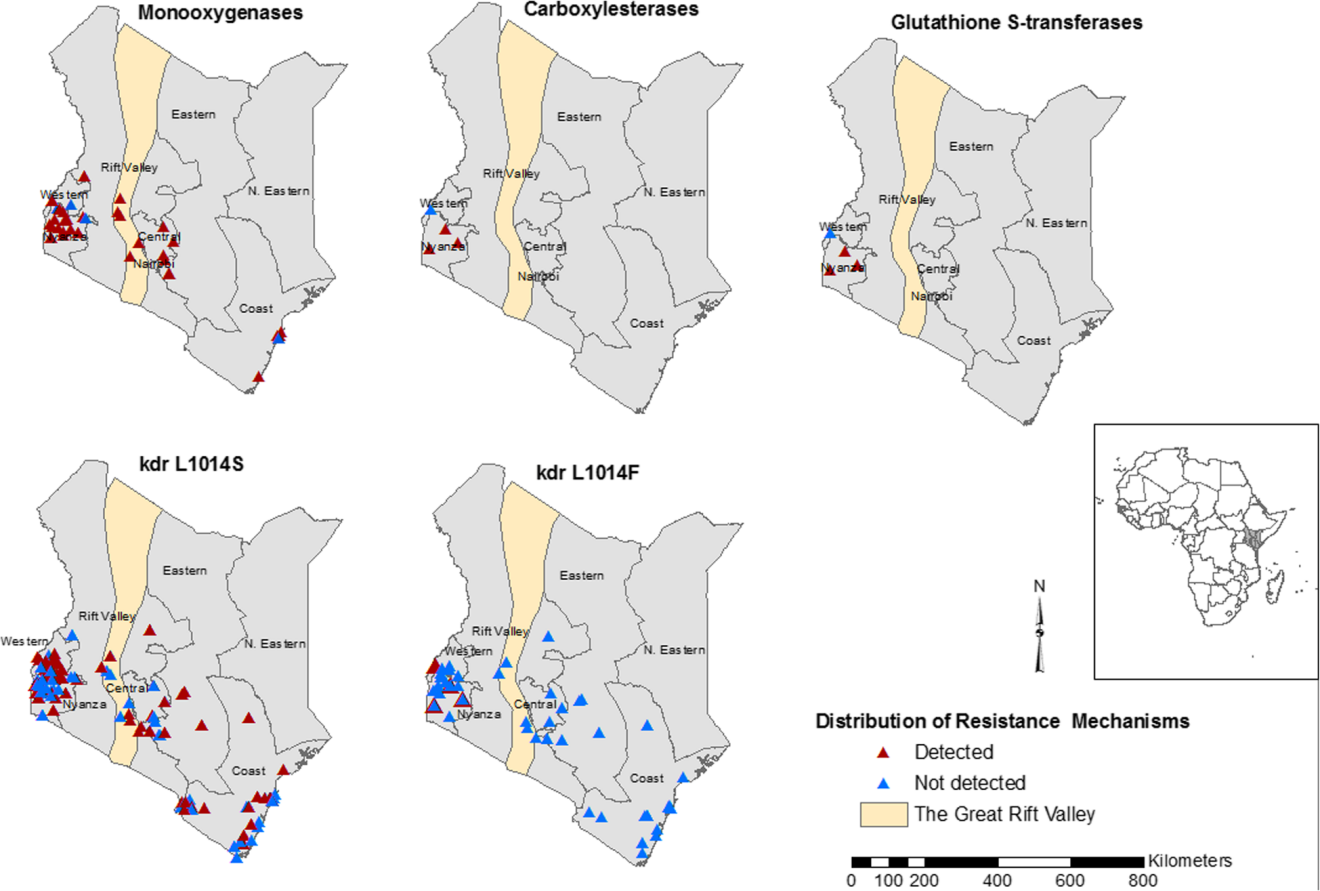
Distribution of resistance mechanisms in *Anopheles* species tested between 1994 and 2015. The *kdr* mutation L 1014S in *Anopheles* species tested is widespread in most regions of Kenya while *kdr* mutation L 1014F has only been detected in the Western region. Elevated activity of monooxygenases in *Anopheles* species tested is widespread in most regions of Kenya while elevated activity of carboxylesterase and glutathione S-transferases tested has only been detected in Western region

The *kdr* mutation L 1014S in *Anopheles* species tested was widespread in the Western region and spreading to the Great Rift Valley, Central, Eastern and Coastal regions (Fig. 2; Additional file 2: Figure S1). The *kdr* mutation L 1014F in *Anopheles* species tested has only been detected in the Western region (Fig. 2; Additional file 2: Figure S2). Similar tests in other regions did not reveal the presence of this mutation. Elevated activity of monooxygenases in *Anopheles* species tested is widespread in the Western region and spreading to the Great Rift Valley, Central, Eastern and Coastal regions (Fig. 2; Additional file 2: Figure S3) whereas elevated activity of carboxylesterase and glutathione S-transferases in *Anopheles* species tested has only been detected in the Western region (Fig. 2; Additional file 2: Figures S4, S5).

## Discussion

The current study has elucidated information on continued occurrence of insecticide resistance in different parts of Kenya that threatens the sustainability of vector control programmes. Consequently, this calls for an urgent need to incorporate annual monitoring of insecticide resistance across the malaria endemic zones in the Kenya Malaria Indicator Survey [47] as per the Global Plan for Insecticide Resistance Management [38]. Although the levels of insecticide resistance may vary across landscapes, even within small geographical scales and at different seasons, the drivers of such variations to insecticide resistance in *Anopheles* population has been attributed to several factors. For example, previous studies [48–50] have linked resistance to selection pressure driven by contamination of malaria vector breeding sites through the application of agricultural pesticides. Kenya, a largely agricultural country is not immune to use of agricultural pesticides that may impact on the performance of insecticides against malaria vectors. For example, carbofuran (carbamate) is used as a pesticide in irrigated rice areas [51] that also provide ideal habitats for mosquito breeding. Furthermore, intensification of insecticide use in public health has also exacerbated the mosquito resistance to pyrethroids. For example, according to surveys conducted by the Kenya Malaria Indicator Survey, LLINs use among the household population has increased from 44% in the 2010 survey to 63% in the 2015 survey [47]. In malaria endemic regions of Kenya such as Western and Coastal regions, the scale-up of ITNs use, and implementation of IRS programs that rely heavily on pyrethroid insecticides may contribute to selection pressure exerted on malaria vectors against pyrethroid-based insecticides [26, 31]. Selection of low susceptible *Anopheles* populations in Kenya could also be driven by migration of resistant malaria vector population from neighbouring regions like Uganda where high resistance to pyrethroids have been reported [9, 31, 52]. Moreover, all these factors may act synergistically rather than in isolation to drive insecticide resistance in the region and may threaten the impact of vector control programmes using these insecticides.

Insecticide resistance to organochlorines, organophosphates and carbamates in Kenya is not widespread largely because their use is limited in Kenya especially in public health. The use of particular organochlorine like DDT is prohibited in any form in the country, and its resistance is only attributed to cross-resistance from pyrethroids and possible population overflow from Uganda into the neighbouring Western region in Kenya [9]. Organophosphates use is limited to agricultural settings against insect pests. Fenitrothion (organophosphate) and carbofuran (carbamate) are used in agricultural systems [51] and could contribute to resistance reported in areas where they are used in agricultural settings [53]. Organophosphates and carbamates have been considered for IRS as an alternative vector control tool against pyrethroids-resistant malaria vectors [8] however sporadic resistance to these insecticides have been reported in Kenya thus justifies the urgent need of an annual resistance monitoring if they are to be considered for IRS in targeted endemic areas.

According to studies reviewed, *kdr* mutation L 1014S was widely reported in *An*. *arabiensis* and *An*. *gambiae* (*s.s*.) in the regions tested in Kenya. This is consistent with earlier studies conducted by Santolamazza et al. [18] that analysed the distribution of *kdr* mutation L 1014S in *An. gambiae* populations and found this mutation to be predominantly found in East African countries [34, 50, 54]. However, knockdown resistance mutation L 1014F was first reported by Ochomo et al. [35] in studies conducted in western Kenya. This mechanism is widespread in West Africa [7, 18, 55] but has recently been reported in eastern African countries; Uganda [56], Ethiopia [57], Sudan [58], Tanzania [59] and more recently in Kenya [35].

The majority of the investigations on the insecticide resistance mechanism were on target site mutations (*kdr*) with a few being on metabolic resistance. The WHO [38] attributes this tendency to the fact that metabolic resistance assays require more stringent testing conditions as fresh mosquitoes are required which are difficult to transport and also are relatively difficult to conduct due to high costs in addition to the interpretation of results that requires strong technical skills. Mosquitoes that have been dead for more than a few minutes at room temperature will have the enzyme levels extremely reduced and degraded hence quantification of enzyme levels may produce inaccurate results [38, 60]. Despite these challenges, more focus should be put on metabolic resistance as evidence has shown that metabolic mechanisms play a bigger role in insecticide resistance than target-site resistance [38].

A large proportion of insecticide resistance mechanism studies, as well as the insecticide susceptibility tests, was mostly focussed on *An*. *gambiae* populations compared to the *An*. *funestus* populations. This propensity to test *An*. *gambiae* as opposed to *An*. *funestus* could be attributed to the difficulty in rearing the progeny of field-collected mosquitoes of this species to obtain large numbers needed to carry out WHO susceptibility tests, biochemical, genetic and molecular tests [61, 62]. Regardless of this difficulty, more information on the insecticide susceptibility and mechanism of *An*. *funestus* population is important to monitor, detect and manage insecticide resistance among this vector species as high level of insecticide resistance has already been reported in South Africa [63]. *Anopheles funestus* is an important vector of malaria in Kenya as it plays an important role in malaria transmission [64–66] and thus warrants insecticide resistance monitoring.

In Africa, there has been an effort to create databases and map the insecticide resistance among the malaria vectors at national or continental scale [18, 39–42]. These databases are necessary to monitor, detect and manage insecticide resistance. In Kenya, the *IRbase* has been consolidated to collate all historical data and current data on insecticide resistance status among malaria vectors in the country. The limitations that were faced while consolidating this database are similar to previous databases that have been generated before [18, 39–42]. In most cases, the studies did not provide geo-coordinates for the study sites thus these sites were geo-referenced using digital geographical databases. A problem encountered with geo-coordinates provided in digital geographical databases is that they are not specifically accurate to the sites where mosquito collections were conducted. From the geospatial maps developed, insecticide resistance data is largely limited to areas with intense malaria vector research that has been conducted over time, and this could be attributed to the proximity to the research institutions. In addition, there have been very few studies in areas where malaria transmission intensity is low. Blank parts in the maps indicated unavailability of published or unpublished sources on insecticide susceptibility and resistance mechanisms from such areas. It is obvious from the maps presented that large areas of the country had no information on insecticide susceptibility and resistance mechanisms among malaria vectors. Thus, there is an urgent need for baseline surveys to be carried out in malaria regions to develop a comprehensive and reliable database for effective insecticide resistance monitoring and management. The *IRbase* generated is easy to use and interpret and will also be easily accessible by the Kenya National Malaria Control Programme and other stakeholders involved in the monitoring and management of insecticide resistance.

Assembling a spatio-temporal defined database of insecticide resistance distribution and insecticide resistance mechanism among malaria vectors in Kenya provides a platform for the future compilation of data. The database was tailored to promote the design of a systematic national monitoring of insecticide resistance while providing a platform for future data sharing. This *IRbase* developed for Kenya will provide an easy guide and accessible information for malaria control managers. The maps generated provide crucial information on the geographical extent of the status of insecticide resistance and insecticide resistance mechanisms for each region in the country. This provides useful information for guiding targeted resource allocation depending on the information needed for each malaria prone area and helps inform decisions to increase insecticide resistance surveillance in areas with little or no information. The *IRbase* may form the basis for determining if there are temporal changes in the insecticide susceptibility and resistance mechanism following vector control interventions. By providing information on the dynamic nature of insecticide resistance, the *IRbase* allows for the planning of appropriate vector interventions strategies where evidence-based decision-making on the appropriate classes of insecticide to be used will be possible. Up to date data from published journal articles and unpublished data will be included in the database yearly for effective monitoring of insecticide resistance and development of appropriate control tools. The *IRbase* generated will be stored at KEMRI-Wellcome Trust Research Programme database and can be accessed by writing to Prof. Charles Mbogo (Cmbogo@kemri-wellcome.org).

## Conclusions

From the available literature examined in this study, phenotypic resistance is observed throughout Kenya with increasing resistance in the Western region of the country. Two mechanisms of resistance, metabolic and *kdr* mutation were identified. Thus, a well-coordinated malaria insecticide resistance database (*IRBase*) in Kenya will help the Kenya National Malaria Control Programme monitor and manage insecticide resistance and assist in the development of improved vector control strategies. Kenya has data on the insecticide resistance currently available, but it is largely limited to areas with intense malaria vector research that has been conducted over time and thus the need to conduct a country-wide analysis in malaria zones to establish the resistance situation. Monitoring and detection of insecticide resistance should be an essential component of all national malaria control efforts to ensure that the most effective vector control methods are being used.

## Additional files

Additional file 1: Figure S1. Malaria vectors susceptibility status against permethrin. **Figure S2.** Malaria vectors susceptibility status against deltamethrin. **Figure S3.** Malaria vectors susceptibility status against lambda-cyhalothrin. **Figure S4.** Malaria vectors susceptibility status against alpha-cypermethrin. **Figure S5.** Malaria vectors susceptibility status against etofenprox. **Figure S6.** Malaria vectors susceptibility status against DDT. **Figure S7.** Malaria vectors susceptibility status against fenitrothion. **Figure S8.** Malaria vectors susceptibility status against malathion. **Figure S9.** Malaria vectors susceptibility status against bendiocarb. **Figure S10.** Malaria vectors susceptibility status against propoxur. (TIFF 9751 kb)

Additional file 2: Figure S1. Status of *kdr* L1014S insecticide resistance mechanism among malaria vectors. **Figure S2.** Status of *kdr* L1014F insecticide resistance mechanism among malaria vectors. **Figure S3.** Status of monooxygenases insecticide resistance mechanism among malaria vectors. **Figure S4.** Status of carboxylesterase insecticide resistance mechanism among malaria vectors. **Figure S5.** Status of glutathione S-transferase insecticide resistance mechanism among malaria vectors. (TIFF 4573 kb)

## Abbreviations

AchE1: Acetylcholinesterase
DDT: Dichlorodiphenyltrichloroethane
DOMC: Division of Malaria Control
GABA: γ-aminobutyric acid
GPS: Global positioning system
GST: Glutathione S-transferases
IRBase: Insecticide resistance database
IRS: Insecticide residual spraying
ITNs: Insecticide-treated nets
*kdr*: Knockdown resistance
KEMRI: Kenya Medical Research Institute
NSE: Non-specific esterases
Rdl: Dieldrin
WHO: World Health Organization
