# Supplementary figures and images for: Current status of insecticide resistance among malaria vectors in Kenya
Source: Parasit Vectors. 2017 Sep 19;10:429. doi: 10.1186/s13071-017-2361-8 (PMC5606043; doi:10.1186/s13071-017-2361-8)

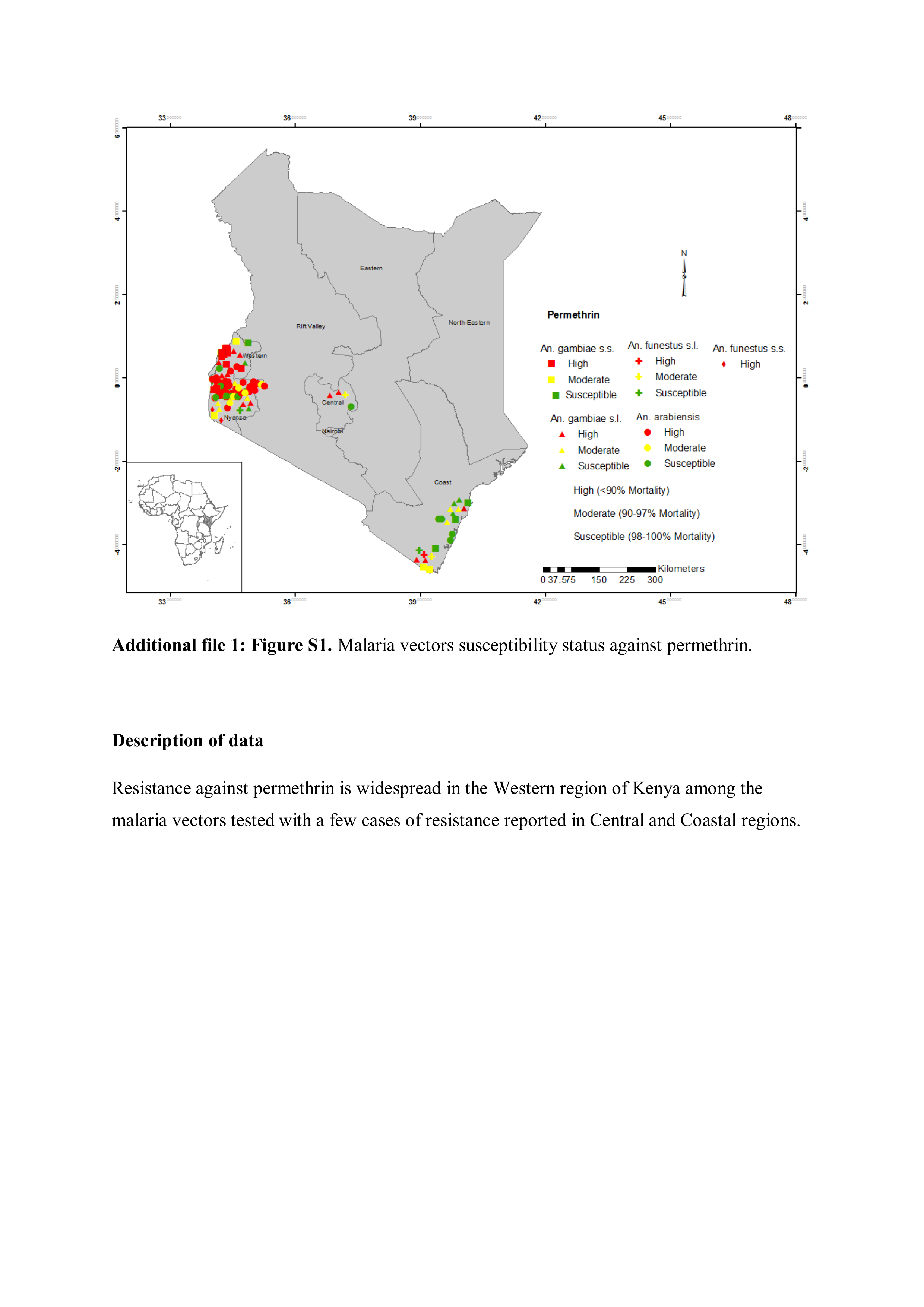

Supplement: Supplementary file 1 — Malaria vectors susceptibility status against permethrin. Figure S2. Malaria vectors susceptibility status against deltamethrin. Figure S3. Malaria vectors susceptibility status against lambda-cyhalothrin. Figure S4. Malaria vectors susceptibility status against alpha-cypermethrin. Figure S5. Malaria vectors susceptibility status against etofenprox. Figure S6. Malaria vectors susceptibility status against DDT. Figure S7. Malaria vectors susceptibility status against fenitrothion. Figure S8. Malaria vectors susceptibility status against malathion. Figure S9. Malaria vectors susceptibility status against bendiocarb. Figure S10. Malaria vectors susceptibility status against propoxur. (TIFF 9751 kb) [file 13071_2017_2361_MOESM1_ESM.tif]

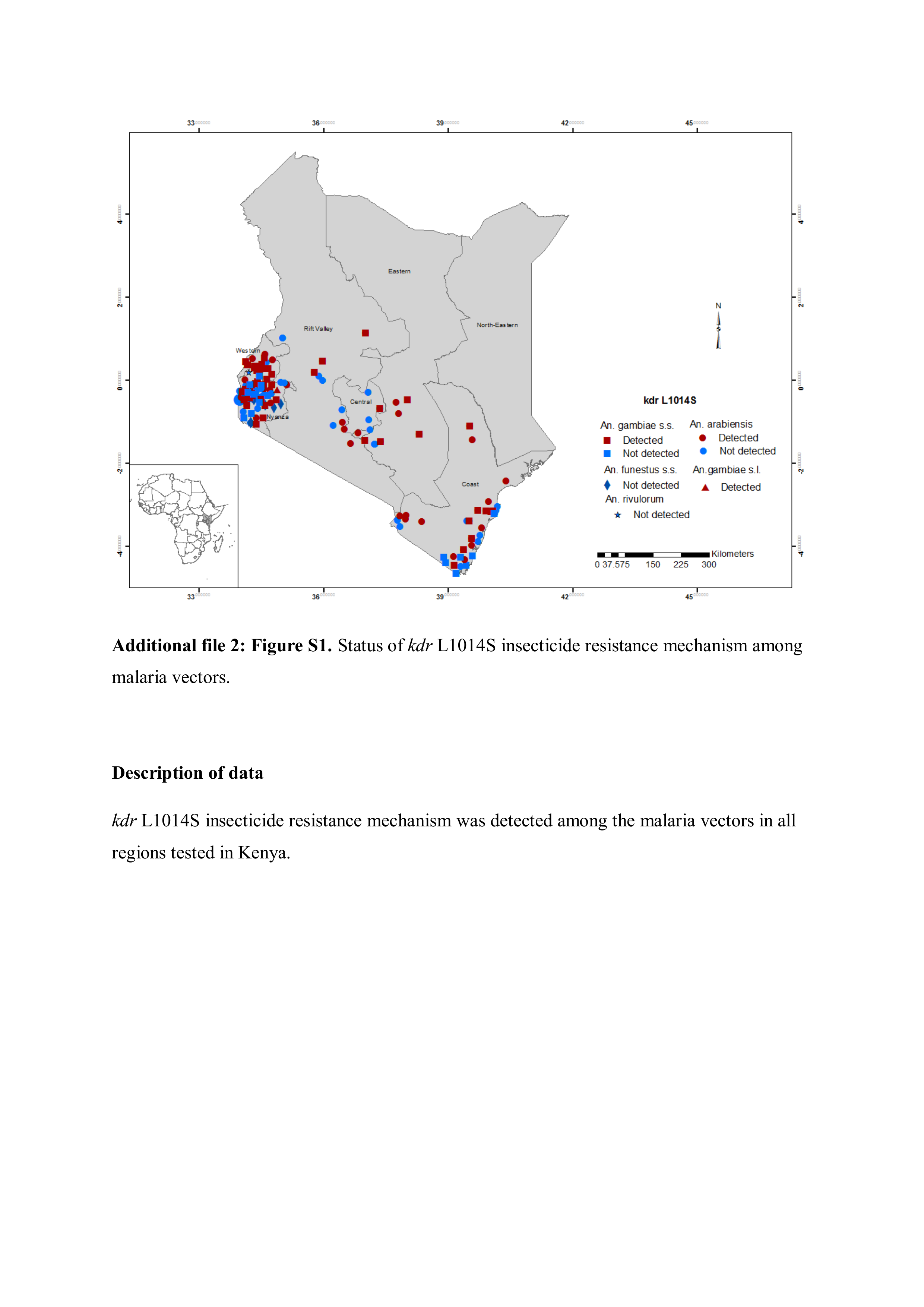

Supplement: Supplementary file 2 — Status of kdr L1014S insecticide resistance mechanism among malaria vectors. Figure S2. Status of kdr L1014F insecticide resistance mechanism among malaria vectors. Figure S3. Status of monooxygenases insecticide resistance mechanism among malaria vectors. Figure S4. Status of carboxylesterase insecticide resistance mechanism among malaria vectors. Figure S5. Status of glutathione S-transferase insecticide resistance mechanism among malaria vectors. (TIFF 4573 kb) [file 13071_2017_2361_MOESM2_ESM.tif]
